# A Comparison of Oxycodone and Alfentanil in Intravenous Patient-Controlled Analgesia with a Time-Scheduled Decremental Infusion after Laparoscopic Cholecystectomy

**DOI:** 10.1155/2016/7868152

**Published:** 2016-09-20

**Authors:** Young Suk Kwon, Ji Su Jang, Na Rea Lee, Seong Su Kim, Young Ki Kim, Byeong Mun Hwang, Seong Sik Kang, Hee Jeong Son, So Young Lim

**Affiliations:** ^1^Department of Anesthesiology and Pain Medicine, College of Medicine, Hallym University, Chuncheon Sacred Heart Hospital, Chuncheon, Republic of Korea; ^2^Department of Anesthesiology and Pain Medicine, College of Medicine, University of Ulsan, Gangneung Asan Hospital, Gangneung, Republic of Korea; ^3^Department of Anesthesiology and Pain Medicine, College of Medicine, Kangwon National University, Kangwon National University Hospital, Chuncheon, Republic of Korea

## Abstract

*Background*. Oxycodone, a semisynthetic opioid, has been widely used for acute and chronic pain.* Objectives*. The aim of this study was to compare the analgesic and adverse effects of oxycodone and alfentanil on postoperative pain after laparoscopic cholecystectomy.* Methods*. This was a prospective, randomized, double-blind study. A total of 82 patients undergoing laparoscopic cholecystectomy were randomly assigned to receive either oxycodone or alfentanil using intravenous patient-controlled analgesia (PCA). PCA was administered as a time-scheduled decremental continuous infusion based on lean body mass for 48 hours postoperatively. Patients were assessed for pain with a visual analogue scale (VAS), the cumulative PCA dose, adverse effects, sedation level at 1, 4, 8, 16, 24, and 48 hours postoperatively, and satisfaction during the postoperative 48 hours.* Results*. There were no significant differences (*p* < 0.05) between the two groups in VAS score, cumulative PCA dose, adverse effects, sedation level at 1, 4, 8, 16, 24, and 48 hours postoperatively, and satisfaction during the postoperative 48 hours.* Conclusions*. Our data showed that the analgesic and adverse effects of oxycodone and alfentanil were similar. Therefore, oxycodone may be a good alternative to alfentanil for pain management using intravenous PCA after laparoscopic cholecystectomy when used at a conversion ratio of 10 : 1. This trial is registered with KCT0001962.

## 1. Introduction

Laparoscopic cholecystectomy (LC) is often accompanied by severe postoperative pain, although it has the advantages of fast recovery and short hospital stay [1]. Postoperative pain management is a critical component of patient care and is associated with patient satisfaction. The major goal of postoperative pain management is to minimize the dose of medications to decrease the side effects, while still providing adequate analgesia [2]. Patient-controlled analgesia (PCA) is among the most popular methods used for postoperative pain control and typically involves the administration of an analgesic agent, most commonly an opioid, using a programmed infusion pump [3].

Oxycodone, a semisynthetic opioid synthesized from thebaine, is used to manage moderate to moderately severe acute or chronic pain. It is a potent *μ*-agonist, with a potency comparable to that of morphine [4]. Many studies have demonstrated good efficacy of oxycodone against postoperative pain [5, 6], and some studies have suggested that oxycodone attenuates visceral pain better than other opioids [5, 7, 8]. Recently, several studies have compared oxycodone and fentanyl in PCA after laparoscopic cholecystectomy [9–11]. Although oxycodone has demonstrated better or similar analgesic effects compared with fentanyl, those studies have shown that a fixed-rate background infusion of oxycodone is associated with a high incidence of adverse effects.

Alfentanil has a rapid onset of action and appropriate pharmacokinetic properties in continuous infusion [12], and it has a lower incidence of nausea and vomiting than fentanyl or sufentanil [13]. For this reason, alfentanil may be a good analgesic in background infusions of PCA. Many clinical studies of PCA have shown that alfentanil with its ease of rapid titration does not cause pulmonary depression/cardiac stress while providing satisfactory analgesia [14].

Intravenous (IV) PCA has not always provided reliable and adequate analgesia. The analgesic concentration in the body might be insufficient for the severity of the early postoperative pain during fixed-rate infusion of the recommended regimen [15]. Fixed higher rates might increase the risk of side effects, such as ventilatory depression. Therefore, we used a time-scheduled decremental continuous infusion to reduce the adverse effects while maintaining analgesic effects by changing the PCA dose over time [16].

Various studies have compared the effects of oxycodone, morphine, and fentanyl on postoperative pain. However, no studies have compared the effects of oxycodone and alfentanil on postoperative pain, and there is no published study of the efficacy and side effects of oxycodone and alfentanil.

In this randomized, prospective, double-blind study, we compared the analgesic and side effects of oxycodone and alfentanil on postoperative pain in patients who were given IV PCA in a time-scheduled decremental mode after laparoscopic cholecystectomy.

## 2. Methods

### 2.1. Study Design

This prospective, randomized, double-blind study was conducted between August 2014 and September 2015. The study protocol was approved by our Institutional Review Board (IRB) and all patients provided informed consent before surgery.

### 2.2. Subject

We enrolled 90 patients who underwent laparoscopic cholecystectomy and belonged to the American Society of Anesthesiologists (ASA) physical status class I or II. Both men and women aged 18–70 years were included. Exclusion criteria were patients who had used preoperative acetaminophen or nonsteroidal anti-inflammatory drugs or opioids, patients who could not describe their pain using the visual analogue scale (VAS), patients who had abnormal liver and kidney function, and pregnant patients.

### 2.3. Methods

We referred to references on the conversion of oxycodone to morphine that suggest a potency ratio of 1 : 1 because there were no recommendations concerning the direct conversion factor of IV oxycodone and IV alfentanil dosages [17, 18]. Alfentanil is approximately ten times more potent than morphine [12]. Therefore, we decided on an alfentanil-to-oxycodone ratio of 1 : 10.

Patients were randomized to either Group A (alfentanil) or Group O (oxycodone) by a simple randomization method using Excel (Microsoft Corp., Seoul, Korea). Group A received IV PCA with 10 mg of alfentanil (Alfenil® 2.5 mg/5 mL, Daewon Pharm, Seoul, Korea), 0.6 mg of ramosetron (Nasea® 0.3 mg/2 mL, Astellas Pharma, Tokyo, Japan), and 76 mL of normal saline. Group O received IV PCA with 100 mg of oxycodone (OxyNorm® 10 mg/1 mL, Mundipharma, Limburg an der Lahn, Germany), 0.6 mg of ramosetron, and 86 mL of normal saline. The rate of PCA, loading dose, and demand dose were calculated based on the lean body mass (LBM), which was calculated using Hume's formula [19].

(1) Men:  LBMkg=0.32810×weightkg+0.33929×heightcm−29.5336Women:  LBMkg=0.29569×weightkg+0.41813×heightcm−43.2933.

Loading doses, demand doses, and background infusion rates were as follows:

(2) Loading  dosemL=LBMkg×0.05 mLDemand  (bolus)  dosemL=LBMkg×0.02 mL.

Background infusion rate (BIR) is described as follows:

(3) First  8-hours  BIR  after  operation mL/h=LBMkg×0.02 mL/h8~24-hours  BIR  after  operation mL/h=LBMkg×0.01 mL/h24~48-hours  BIR  after  operation mL/h=LBMkg×0.005 mL/h.

Doses have been rounded to the second decimal place. The time-scheduled decremental infusion mode was performed using a PCA device (Accumate 1100®, Woo Young Medical, Jincheon, Korea) automatically.

The patients provided informed consent on the day before the operation and were instructed on the use of PCA. Anesthesia was performed in the same manner in both groups and all patients received 0.2 mg of intramuscular (IM) glycopyrrolate 30 minutes preoperatively. After entering the operating room, patients were monitored using standard monitoring devices. Anesthesia was induced with 2 mg/kg of propofol and 0.6 mg/kg of rocuronium and maintained with desflurane and N_2_O. Then, 0.3 mg of ramosetron was injected intravenously and a loading dose of PCA was administered 15 minutes before the end of the operation. Immediately after the loading doses, the background infusion was started. Neuromuscular blockade was reversed with 0.4 mg of glycopyrrolate and 10 mg of pyridostigmine. After surgery, patients were extubated, provided their vital signs were within normal limits, and were transferred to the postanesthesia care unit.

### 2.4. Assessment

Pain level, cumulative doses, sedation scale, and adverse effects were measured at 1, 4, 8, 16, 24, and 48 hours postoperatively. The severity of the patients' pain was evaluated using the visual analogue scale (VAS), with scores ranging from 0 (no pain) to 10 (worst pain possible). The VAS was evaluated when coughing and resting. Sedation was scored using the Inova Sedation Scale (ISS): 1, alert; 2, occasionally drowsy, easy to rouse; 3, dozing intermittently; 4, asleep, easy to wake; 5, difficult to wake; 6, unresponsive [20].

Any nausea, vomiting, dizziness, headache, respiratory depression, pruritus, or difficulty voiding was recorded as an adverse effect. The patient's satisfaction with PCA during the 48 hours postoperatively was assessed according to the following scale: 1, very satisfactory; 2, satisfactory; 3, neutral; 4, unsatisfactory; 5, very unsatisfactory.

### 2.5. Statistical Analysis

Data are expressed as the mean ± standard deviation (SD). The demographic data of the two groups were analyzed with Student's *t*-test and chi-square test. Comparison analysis of the incidence of postoperative nausea, vomiting, and other adverse effects was performed using the chi-square test and Fisher's exact test. The cumulative PCA dose and the sedation scale were assessed using the Mann-Whitney *U* test, while the VAS was assessed by repeated-measures analysis of variance (ANOVA). The satisfaction score of the two groups was assessed via the chi-square test. A probability of <0.05 was considered to indicate statistical significance. All data were analyzed using SPSS ver. 22 (SPSS, Chicago, IL, USA).

Based on previous similar studies, the sample size was 41 patients per group. The cumulative PCA dose difference between the two groups was 5 mL and the standard deviation was 8.0 with power of 80% and *α* = 0.05. Therefore, we enrolled 90 subjects into our study considering 10% as the exclusion rate.

## 3. Results

**Figure 1:**
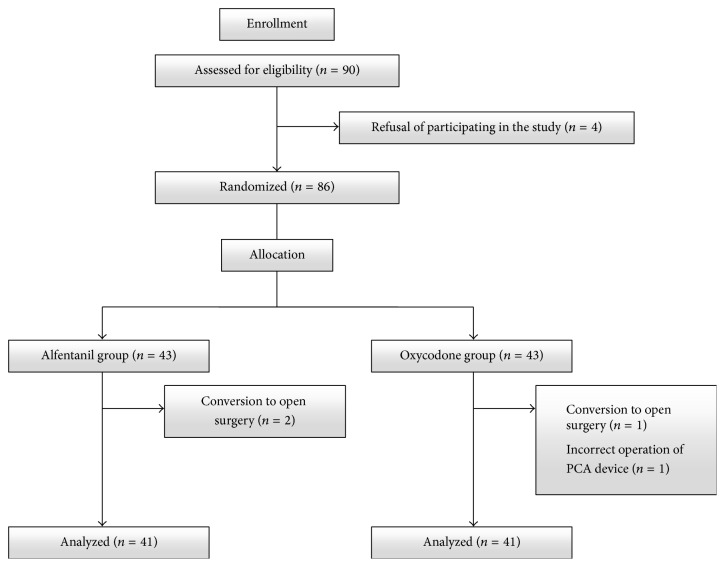
CONSORT flowchart.

90 patients were assessed for eligibility. Four patients declined to participate in the study, and 86 patients were randomized to treatment with alfentanil (*n* = 43) or oxycodone (*n* = 43). Two patients in Group A and one patient in Group O were excluded because they ultimately underwent open laparotomy. One patient in Group O was excluded due to incorrect operation of the PCA device. In total, 41 patients were left for analysis in each group (Figure 1). There were no significant differences between the two groups with regard to age, sex, height, weight, LBM, or operation time (Table 1).

**Figure 2:**
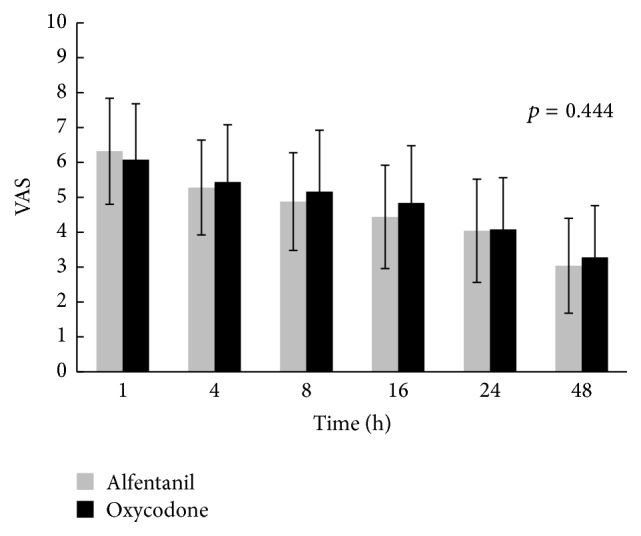
Visual analogue scale of pain during coughing between 1 and 48 h after the operation. Means and standard deviation are shown. *p* values were calculated by repeated-measures analysis of variance. There were no significant differences between two groups (*p* value < 0.05). VAS: visual analogue scale.

**Figure 3:**
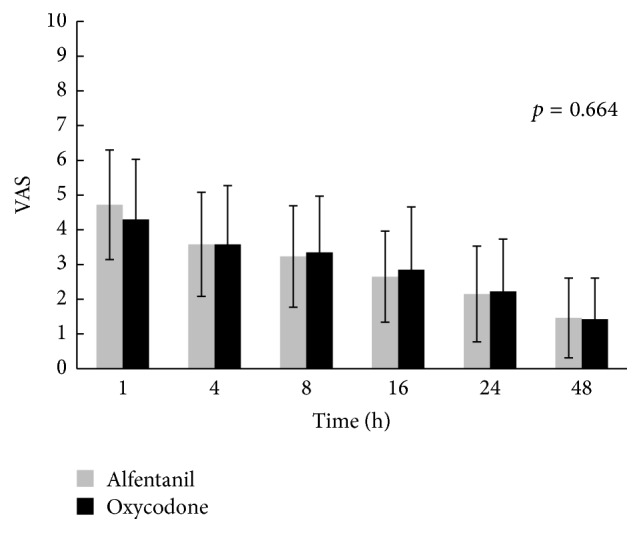
Visual analogue scale of pain at resting between 1 and 48 h after the operation. Means and standard deviation are shown. *p* values were calculated by repeated-measures analysis of variance. There were no significant differences between two groups (*p* value < 0.05). VAS: visual analogue scale.

There was no significant difference in the VAS when resting or coughing between the two groups at 1, 4, 8, 16, 24, and 48 h postoperatively (Figures 2 and 3).

**Figure 4:**
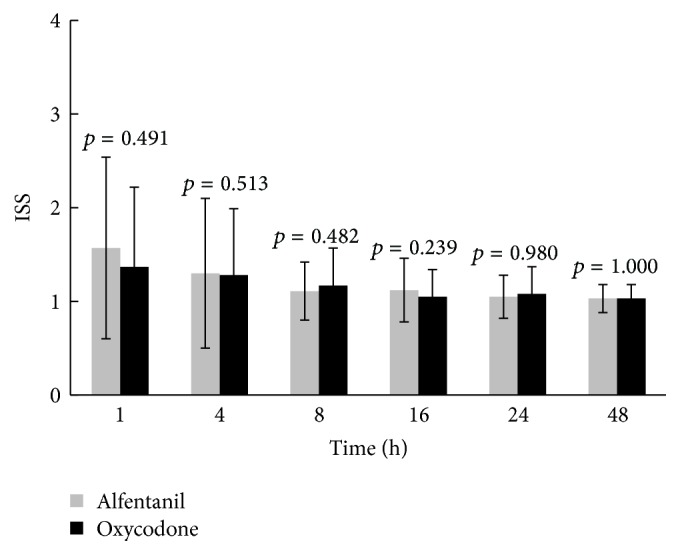
Inova Sedation Scale of patients between 1 and 48 h after the operation. Means and standard deviation are shown. *p* values were calculated by Mann-Whitney *U* test. There were no significant differences between two groups (*p* value < 0.05). ISS: Inova Sedation Scale.

The incidence of postoperative nausea and vomiting in Group O was not significantly different from Group A at 1, 4, 8, 16, 24, and 48 h postoperatively (Table 2). Regarding adverse effects such as headache, dizziness, respiratory depression, voiding difficulty, and pruritus, there were no differences between Group A and Group O at 1, 4, 8, 16, 24, and 48 h postoperatively (Table 3). Sedation grade was expressed using the ISS, and there were no differences between the two groups at 1, 4, 8, 16, 24, and 48 h postoperatively (Figure 4). In addition, there was no difference in satisfaction during the 48 h postoperatively between the two groups (Table 4).

**Figure 5:**
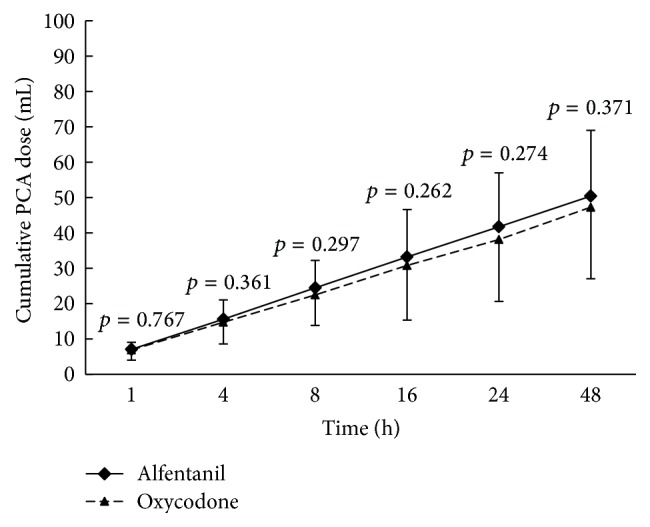
Cumulative patient-controlled analgesia dose measured between 1 and 48 h after the operation. Means and standard deviation are shown. *p* values were calculated by Mann-Whitney *U* test. There were no significant differences between two groups (*p* value < 0.05). PCA: patient-controlled analgesia.

The cumulative PCA dose of the 2 groups was not significantly different at 1, 4, 8, 16, 24, and 48 h postoperatively (Figure 5).

## 4. Discussion

Oxycodone was first synthesized from thebaine in 1916, and it is now one of the most widely used opioids for pain management [21–23]. Oxycodone is as potent as morphine but has superior analgesic effects over both morphine and placebos regarding mechanical and thermal noxious stimuli of the esophagus [5–7]. Therefore, oxycodone may be more effective than other opioids at equianalgesic dosages after surgery in which the visceral pain component is a large contributor to a patient's overall postoperative pain [7, 24]. Oxycodone had similar and sometimes better effects in postoperative analgesia compared to fentanyl [11]. Koch et al. [6] compared the effects of intravenous oxycodone and fentanyl on postoperative visceral pain after outpatient laparoscopic cholecystectomy and found that oxycodone provided better analgesia but also had more side effects.

Alfentanil hydrochloride, a derivative of fentanyl, is a potent analgesic characterized by a quick onset time, short duration of action, low toxicity, and short elimination time [25, 26]. The advantage of alfentanil over other opioids is the short recovery time and its use for PCA has been described for postoperative analgesia [27]. With respect to rapid recovery, alfentanil is superior to fentanyl [28], but the speed of recovery is associated with a reduction in the postoperative duration of analgesia. Therefore, a concurrent infusion of alfentanil may be necessary for optimum analgesia because postoperative pain is generally more constant and of longer duration.

Considering the lack of guidelines for the direct conversion dose ratio of intravenous oxycodone to intravenous alfentanil, this study reviewed previous research in order to identify the used ratios. Parenteral oxycodone appears to be equipotent to morphine [17]. The potency of alfentanil is approximately 10 times that of morphine [12]. On that basis, we calculated a workable alfentanil-to-oxycodone ratio of 1 : 10. In this study, Group O showed no significant difference in the cumulative PCA dose, while maintaining similar VAS values comparable to that of Group A. Therefore, oxycodone had comparable effects for pain relief compared to alfentanil when used at a conversion ratio of 10 : 1 in our study.

Postoperative pain can cause many adverse effects such as atelectasis, prolonged hospital stay, and decreased patient satisfaction. A background infusion of PCA can improve the level of analgesia and reduce breakthrough pain in the postoperative period [29]. However, a routine fixed-rate background infusion increases the analgesic dosage and the incidence of adverse respiratory events in the postoperative period. With a routine fixed background infusion of oxycodone, postoperative nausea and vomiting (PONV) was common and relatively long-lasting [10, 11]. Therefore, it is necessary to establish the infusion method while varying the infusion rates of the analgesics. We devised a study to reduce the adverse effects, while maintaining analgesic effects, by changing the PCA dose over time. Kim et al. [16] reported that a time-scheduled decremental continuous infusion provided sufficient analgesic effect without increasing side effects. In this study, we compared oxycodone and alfentanil in terms of the adequacy of postoperative pain control using a time-scheduled decremental mode IV PCA in patients who underwent laparoscopic cholecystectomy. The intensity of postoperative pain depends on the type of surgery performed [30]. Our preliminary study found that the pain grade without PCA was the highest until 6 to 8 hours after laparoscopic cholecystectomy and decreased significantly after 24 hours postoperatively. Considering these changes, in this study, we used the time-scheduled continuous infusion method to reduce the flow rate of the background infusion at 8 and 24 hours postoperatively.

We also used opioids based on the LBM to reduce adverse effects in this study. Many opioid pharmacokinetic parameters such as clearance are considered to be more closely related to lean body mass [31]. Obesity has been demonstrated to prolong the elimination half-life of alfentanil [32]. Oxycodone has a duration of action similar to morphine but lower clearance [33], and the duration of action can be prolonged in obese patients. As a result, in an obese patient, total body weight-based dosing may increase the incidence of adverse effects compared with lean body mass-based dosing. Therefore, opioid dosages based on LBM rather than total body weight may be more accurate. Therefore, we administered doses based on LBM using Hume's method in this study.

Nausea and vomiting are common postoperative adverse effects in PCA. Opioids stimulate the chemoreceptor trigger zone in the medulla to cause nausea and vomiting, which decrease overall patient satisfaction. Also, nausea and vomiting may worsen the severity of incisional pain. Many patients have reported that postoperative vomiting is more unpleasant than postoperative pain [34]. Several studies that used a routine fixed background infusion of oxycodone found a notably higher incidence of nausea, which did not decrease significantly over time [10, 11], while alfentanil caused less postoperative nausea and vomiting (PONV) than equipotent doses of fentanyl or sufentanil in outpatients [13]. In our study, however, oxycodone did not differ significantly from alfentanil and had a low incidence of nausea. In the first postoperative hour, the incidence of nausea in Groups A and O was relatively higher than in other periods. This would include the effects of a loading dose given before the end of the operation, the inhalation agent used during the operation, and laparoscopic surgery. Another factor may be the time-scheduled decremental infusion because of the administration of a high opioid concentration given several hours postoperatively. Regarding the incidence of vomiting, three patients in Group A experienced vomiting whereas no patients in Group O did, but this difference was not statistically significant. Several studies have demonstrated good efficacy of the antiemetic ramosetron against PONV after laparoscopic surgery [35–37]; we opted to use ramosetron postoperatively in our study patients. Without this drug, the incidence of PONV would have been higher in this study.

Patients experienced dizziness more frequently in Group O (13 patients) than Group A (6 patients), but the difference was not statistically significant. One patient in each group experienced urinary retention. Although the incidence was very low and statistically insignificant, both patients also complained of lower abdominal discomfort and severe incisional pain concurrent with urinary retention.

The VAS score decreased gradually over time and changes of VAS are not different over time when resting and when coughing in both groups. However, one patient in each group had flatulence and was not discharged until 24 hours postoperatively. Both patients complained of abdominal discomfort and maintained a high VAS score until discharge. Opioids tend to inhibit intestinal propulsion and increase gut transit time, which can lead to postoperative ileus [38]. Therefore, careful monitoring and management of flatulence are essential to lessen postoperative pain.

There are many studies comparing the effects of different opioids on acute postoperative pain. To the best of our knowledge, no previous study has compared intravenous oxycodone and intravenous alfentanil. We hypothesized that alfentanil would be superior to oxycodone in terms of the adverse effects of postoperative PCA. However, alfentanil and oxycodone did not differ significantly in terms of pain control and adverse effects in our study. These results may indicate that oxycodone had comparable effects compared to alfentanil at a conversion ratio of 10 : 1 with a time-scheduled decremental infusion mode of PCA based on the LBM. Therefore, using a conversion factor of 10 : 1, oxycodone is a useful alternative to alfentanil for IV PCA after laparoscopic cholecystectomy. Further studies in various clinical settings will be needed to determine the adequate potency ratio.

## 5. Conclusions

Intravenous oxycodone produces similar analgesic and adverse effects to intravenous alfentanil in the treatment of postoperative pain after laparoscopic cholecystectomy when used at a conversion ratio of 10 : 1. Based on these results, we conclude that oxycodone may be used as a good alternative to alfentanil in pain management after laparoscopic cholecystectomy without increasing adverse effects.

## Figures and Tables

**Table 1:** Demographic characteristics of patients.

Characteristics	Alfentanil group (*N* = 41)	Oxycodone group (*N* = 41)	*p* value
Age (years)	46.2 ± 12.8	46.4 ± 13.8	0.588
Sex (male/female, *N* (%))	16 (39)/25 (61)	14 (34.1)/27 (65.9)	^*∗*^0.647
Height (cm)	163.0 ± 7.8	162.4 ± 9.0	0.411
Weight (kg)	67.8 ± 11.9	67.9 ± 12.7	0.677
LBM (kg)	48.5 ± 7.2	48.1 ± 8.1	0.372
OP time (minutes)	44.6 ± 19.2	42.8 ± 16.7	0.174

LBM: lean body mass; OP: operation.

All data are expressed as mean ± standard deviation or number (percentage). The data were analyzed using Student's *t*-test and ^*∗*^chi-square test. There were no significant differences between two groups (*p* value < 0.05).

**Table 2:** Incidence rate of postoperative nausea and vomiting.

Time after operation	Alfentanil group (*N* = 41)		Oxycodone group (*N* = 41)		*p* value (N/V)
	Nausea	Vomiting	Nausea	Vomiting	
<8 h	14 (34.1%)	2 (4.9%)	8 (19.5%)	0 (0%)	^*∗*^0.135/0.494
<1 h	8 (19.5%)	1 (2.4%)	7 (17.1%)	0 (0%)	^*∗*^0.775/1.000
1~4 h	5 (12.2%)	1 (2.4%)	2 (4.9%)	0 (0%)	0.432/1.000
4~8 h	5 (12.2%)	0 (0%)	3 (7.3%)	0 (0%)	0.712/—

8~24 h	2 (4.9%)	1 (2.4%)	5 (12.2%)	0 (0%)	0.432/1.000
8~16 h	2 (4.9%)	1 (2.4%)	3 (7.3%)	0 (0%)	1.000/1.000
16~24 h	0 (0%)	0 (0%)	3 (7.3%)	0 (0%)	0.241/—

24~48 h	0 (0%)	0 (0%)	1 (2.4%)	0 (0%)	0.494/—

N: nausea; V: vomiting.

Values are expressed as number of patients (percentage). The data were analyzed using Fisher's exact test and ^**∗**^chi-square test. There were no significant differences between two groups (*p* value < 0.05).

**Table 3:** Incidence rate of postoperative adverse effects.

Incidence of adverse effects	Alfentanil group (*N* = 41)	Oxycodone group (*N* = 41)	*p* value
Dizziness	6 (14.6%)	13 (31.7%)	^*∗*^0.067
Headache	1 (2.4%)	2 (4.9%)	1.000
Respiratory depression	1 (2.4%)	1 (2.4%)	1.000
Pruritus	0 (0%)	1 (2.4%)	1.000
Urinary retention	1 (2.4%)	1 (2.4%)	1.000

Values are expressed as number of patients (percentage). The data were analyzed using Fisher's exact test and ^**∗**^chi-square test. There were no significant differences between two groups (*p* value < 0.05).

**Table 4:** Satisfaction of patients at the postoperative 48 hours.

Satisfaction	Alfentanil group (*N* = 41)	Oxycodone group (*N* = 41)
Very satisfied	14 (34.1%)	17 (41.5%)
Satisfied	17 (41.5%)	15 (36.6%)
Neutral	8 (19.5%)	6 (14.6%)
Dissatisfied	2 (4.9%)	3 (7.3%)
Very dissatisfied	0 (0%)	0 (0%)

Values are expressed as number of patients (percentage). The data were analyzed using chi-square test (*p* value = 0.710).
